# PReS-FINAL-2246: CBCT versus orthopan tomogram detecting TMJ alteration in JIA

**DOI:** 10.1186/1546-0096-11-S2-P236

**Published:** 2013-12-05

**Authors:** P Arvonen, J Naujokaityte, M Arvonen, J Niinimäki, P Vähäsalo, P Pirttiniemi

**Affiliations:** 1 Department of Dentistry, Oulu University, Oulu, Finland; 2 Pediatrics, Kuopio University Hospital, Kuopio, Finland; 3 Radiology, Oulu University Hospital, Oulu, Finland; 4 Pediatrics, Oulu University Hospital, Oulu, Finland

## Introduction

TMJ is frequently affected in JIA, but there is no data comparing imaging techniques in detecting TMJ bone changes.

## Objectives

The purpose was to compare the usefulness of CBCT and OPG in detecting condylar changes, including erosion, flattening and mandibular asymmetry in children with JIA.

## Methods

61 JIA patients with TMJ alterations were imaged by OPG and CBCT (mean age 11 years, 5-17 years). Mandibular ramus height was measured on OPG (distance between point Condylion and Gonion) and condyle height on CBCT (distance between incisura and point Condylion). The difference between right and left sides was calculated. Condylar changes were graded in five damage scores (Billiau 2007) by two dentists.

## Results

CBCT was superior to OPG in inter-observer reproducibility in Billiau classification (r^2 ^= 0.835 vs. r^2 ^= 0.64, 122 joints). Classification correlated between OPG and CBCT (R = 0.637, p < 0.001). OPG was superior to CBCT in repeatability of measurement of asymmetry of ramus or condyle heights (r^2^=0.921 vs. 0.766). Asymmetry during opening of mouth was correlating better with OPG than CBCT asymmetry measurements (R = -0.624, vs. -0.519) and they were correlating (0.742, p < 0.001).

## Conclusion

OPG is a valid method in detecting asymmetry of mandibular ramus lengths in JIA patients. CBCT is superior to OPG in detecting bony TMJ changes because it detects three dimensionally the bony structures with accuracy. In CBCT there are more sources of errors in detecting the condyle height.

## Disclosure of interest

None declared.

